# Isolation of a Novel Microcystin-Degrading Bacterium and the Evolutionary Origin of *mlr* Gene Cluster

**DOI:** 10.3390/toxins11050269

**Published:** 2019-05-13

**Authors:** Lian Qin, Xiaoxing Zhang, Xiaoguo Chen, Ke Wang, Yitian Shen, Dan Li

**Affiliations:** School of Resource and Environmental Engineering, Wuhan University of Technology, 122 Luoshi Road, Wuhan 430070, China; qinlian8@gmail.com (L.Q.); nancy-zhang@whut.edu.cn (X.Z.); wang-ke@whut.edu.cn (K.W.); shiyitian@126.com (Y.S.); lid_123@126.com (D.L.)

**Keywords:** microcystin, degradation, *mlr* gene cluster, evolutionary origin, mechanism, *Sphingopyxis*

## Abstract

The *mlr*-dependent biodegradation plays an essential role in the natural attenuation of microcystins (MCs) in eutrophic freshwater ecosystems. However, their evolutionary origin is still unclear due to the lack of *mlr* gene cluster sequences. In this study, a *Sphingopyxis* sp. strain X20 with high MC-degrading ability was isolated, and the *mlrA* gene activity was verified by heterologous expression. The whole sequence of the *mlr* gene cluster in strain X20 was obtained through PCR and thermal asymmetric interlaced (TAIL)-PCR, and then used for evolutionary origin analyses together with the sequences available in GenBank. Phylogenetic analyses of *mlr* gene clusters suggested that the four *mlr* genes had the same origin and evolutionary history. Genomic island analyses showed that there is a genomic island on the genome of sphingomonads that is capable of degrading MCs, on which the *mlr* gene cluster anchors. The concentrated distribution of the *mlr* gene cluster in sphingomonads implied that these genes have likely been present in the sphingomonads gene pool for a considerable time. Therefore, the *mlr* gene cluster may have initially entered into the genome of sphingomonads together with the genomic island by a horizontal gene transfer event, and then become inherited by some sphingomonads. The species other than sphingomonads have likely acquired *mlr* genes from sphingomonads by recently horizontal gene transfer due to the sporadic distribution of MC-degrading species and the *mlr* genes in them. Our results shed new light on the evolutionary origin of the *mlr* cluster and thus facilitate the interpretation of characteristic distribution of the *mlr* gene in bacteria and the understanding of whole *mlr* pathway.

## 1. Introduction

Harmful cyanobacterial blooms (HCBs) in freshwater bodies have become a global environmental problem, and are receiving growing attention with their increase in magnitude, frequency, and duration [1]. Microcystins (MCs) are a group of cyclic heptapeptide hepatotoxins produced by HCBs, among which microcystin-LR (MCLR) is the most widespread and best studied [2,3]. Due to their hepatotoxicity and potential carcinogenic activity, the World Health Organization (WHO) has suggested a guideline value of 1 µg·L^−1^ MCLR equivalents for drinking water [4].

MCs are resistant to traditional water treatment processes due to their chemically stable structure [5]. Biodegradation is one of the major pathways for MCs’ attenuation in the natural environment, and thus may be used in the removal of MCs [3,6]. Although a variety of MC-degrading pathways have been proposed, only the *mlr*-dependent pathway is confirmed and elucidated in detail. In this pathway, four genes—*mlrA*, *mlrB*, *mlrC* and *mlrD*—are involved, which form an *mlr* gene cluster and encode three hydrolysis enzymes (MlrA, MlrB, and MlrC) and one oligopeptide transporter-like protein (MlrD), respectively [7]. MlrA is the first enzyme involved in MCLR degradation, which hydrolyzes the circular MCLR into linear MCLR. Then, the MlrB enzyme hydrolyzes the linear MCLR into a tetrapeptide. MlrC is responsible for the further hydrolysis of the tetrapeptide to amino acids and smaller peptides [8]. Later studies showed that MlrC can also decompose linear MCLR to Adda directly [9,10]. This is the most well-characterized pathway for MC degradation thus far. However, there are still some questions to be answered about this process. Currently, only the part of the process from MCLR to Adda has been elucidated [11]; how the Adda residue is decomposed is still unclear, and the gene involved in Adda degradation is also unknown. The Adda residue is a specific amino that is only found in MCs and nodularins to date. Besides, it is a main building block for the synthesis of MCs, and is crucial to the toxicity of MCs [12]. Understanding the metabolism of Adda is essential for the ecological risk assessment of MCs. In addition, the function of MlrD has not been determined, although it is deduced to be responsible for the transport of MCLR or its products [13].

Up to now, over 70 strains of MC-degrading bacteria have been isolated from various environmental habitats, and the majority of them are from phylum proteobacteria, especially from the class α-proteobacteria [13,14,15,16,17]. Most of these α-proteobacteria are proved to harbor the *mlrA* gene, suggesting that they may degrade MCs through the *mlr*-dependent mechanism [18,19]. In addition, these strains have been found in many places over the world, and most of them possess strong MC-degrading activity [18,19,20,21,22]. These findings imply that MC-degrading bacteria harboring *mlr* genes may play a significant role in the diminishment of MCs in the natural environment, and may be applied to MCs pollution treatment. However, by now, we know little about the diversity of *mlr* genes. Moreover, acknowledgement of the distribution of the *mlr*-dependent pathway in bacteria is also deficient. These deficiencies affect the assessment of MCs pollution negatively, and impede the application of biodegradation process to MCs pollution treatment.

Understanding the evolutionary origin of *mlr* genes will help to infer the diversity of *mlr* genes and the distribution of the *mlr*-dependent pathway among bacteria. It also conduces determining the degradation mechanism adopted by novel MC-degrading isolates [13]. However, very few studies have been performed on the evolutionary origin of *mlr* genes, and the existing results are controversial. The sporadic distribution of *mlrA* genes in *Sphingomonas* supported that *Sphingomonas* may acquire the *mlrA* gene by horizontal gene transfer [23], whereas phylogenetic analyses argued that *mlrA* genes are likely as conserved and ancient as the 16S rRNA gene [20]. More recently, Zhu [13] investigated the evolutionary origin of *mlrA* through comparing the *mlrA* tree with the 16S rDNA tree. The congruent topologies in both trees for α-proteobacteria and the incongruent topologies for other proeobacteria indicated that a-proteobacteria is likely to have acquired the *mlrA* gene by vertical evolution, and other proeobacteria possibly have acquired the *mlrA* gene by horizontal gene transfer [13]. Although this thesis can explain the distribution and diversity of *mlrA* among MC-degrading bacteria, it is inferred based on limited sequences [13]. The confirmation of this will have to await additional MC-degrading isolates and their *mlr* gene sequences. Furthermore, only the evolutionary origin of the *mlA* gene has been investigated currently. The origin of the other three genes involved in this process—*mlrB*, *mlrC* and *mlrD*—is still unknown, and whether these genes had the same origin and evolutionary history with *mlrA* is unclear.

In this study, a novel *Sphingopyxis* sp. strain X20 with high MCs degradability was isolated from Dianchi Lake sediment. The whole *mlr* gene cluster was sequenced by PCR and TAIL-PCR, and the activity of the *mlrA* gene was verified by heterologous expression. To clarify the evolutionary origin of the *mlr* gene cluster, a spliced sequences dataset was constructed based on the sequences of four *mlr* genes from each isolate available in GenBank. The evolutionary origin of the *mlr* gene cluster was deduced through phylogenetic analyses of the spliced sequences, *mlrA* sequences, and the related 16S rDNA sequences. Genomic island (GI) analyses of sphingomonads and the strain X20 were also conducted to further verify the origin of the *mlr* gene cluster.

## 2. Results and Discussion

### 2.1. Isolation and Degradation Activity of MC-Degrading Bacterium

**Figure 1:**
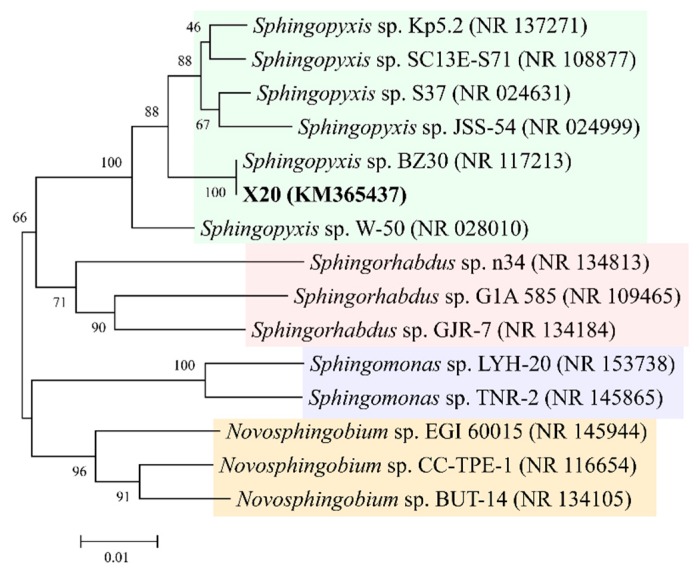
Phylogenetic analysis of the 16S rDNA sequences from strain X20 and the related type strains by the neighbor-joining (NJ) method in MEGA7. Bootstrap values represent percentages from 1000 replicates of the data.

An MC-degrading bacterium, which was designated as strain X20, was isolated from the sediments of Dianchi Lake. It was a gram-negative aerobic bacterium, and formed bright yellow, round colonies on solid yeast extract-peptone medium (YPM). To identify it, a 16S rDNA tree was constructed using sequences of strain X20 and the related type strains (Figure 1). Strain X20 had the highest homology with *Sphingopyxis* sp. BZ30 in the tree, with 100% bootstrap support. Moreover, they clustered with five other types of *Sphingopyxis* to form a clade, which was clearly separated from other genera (Figure 1). These results suggested that strain X20 belongs to the genus *Sphingopyxis*.

**Figure 2:**
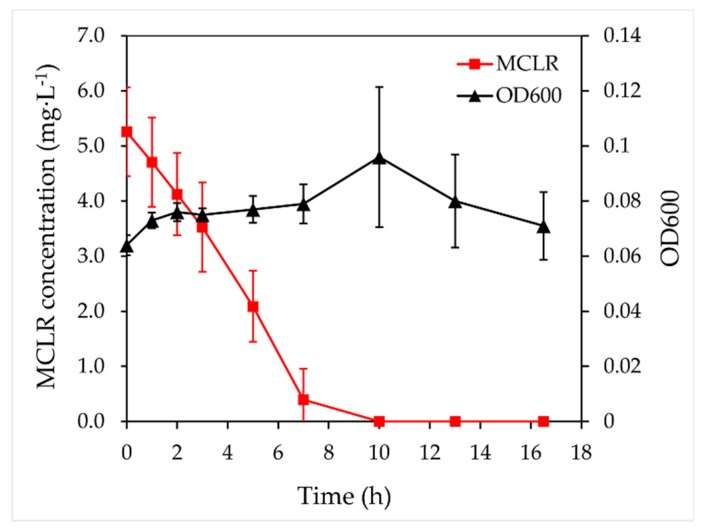
Degradation of microcystin-LR (MCLR) by strain X20 at 30 °C. Error bars represent the standard deviations.

Strain X20 degraded 5 mg·L^−1^ of MCLR to below the detection limit without a lag phase within 10 h (Figure 2). The pseudo-first order rate constant was up to 0.22 h^−1^, which was higher than that of most MC-degraders currently isolated [13,14,16]. This high rate suggested that strain X20 may be one of the main degraders involved in MCs degradation in Dianchi Lake. Their appearance may explain why the concentration of MCs in Dianchi Lake has been maintained at a relatively lower level, although the toxic cyanobacterial bloom frequently occurs [24]. The high rate also implied that these indigenous bacteria have the potential to be used for the treatment of MCs pollution.

Besides strain X20, many other species of sphingomonads, which comprises five closely related genera—*Sphingopyxis*, *Sphingomonas*, *Sphingosinicella*, *Novosphingobium* and *Sphingobium*—have also been found to degrade MCs [20,21,22,25,26,27]. These species were isolated from many different environmental habitats around the world, and usually have strong MC-degrading ability, suggesting that they may play an important role in the natural attenuation of MCs. Sphingomonads are a versatile bacteria group previously classified as *Sphingomonas* [28]. They are widely distributed in both polluted and unpolluted environments. In addition to MCs, sphingomonads can decompose a variety of hazardous organic compounds, such as polycyclic aromatic hydrocarbons, dioxins, herbicides, and pesticides [14,28]. Catabolic diversity may provide them a competitive advantage over other bacteria and help them acclimate to diverse environments. This may explain the frequent appearance of sphingomonads that are capable of degrading MCs in eutrophic waterbodies [29].

### 2.2. Whole mlr Gene Cluster in Strain X20

The whole sequence of the *mlr* gene cluster in strain X20 was successfully amplified and sequenced by using traditional PCR and thermal asymmetric interlaced (TAIL)-PCR approaches. The activity of the *mlr* gene cluster obtained was verified through the heterologous expression of *mlr*A gene in *E. coli*. Biodegradation experiments showed that both the recombinant strains and the recombinant enzyme can degrade MCLR (data not shown). During the degradation of MCLR, a linear MCLR, the same intermediate product as that produced by ACM-3962 [8], occurred and accumulated in culture media. These results demonstrated that the same *mlr*-dependent pathway is adopted in strain X20 as other *Sphingopyxis* MC-degraders [9,21]. Previous results indicated that MlrD may be responsible for the transportation of MCs during degradation, whereas our result showed that MlrD is not essential in the first step of MCLR degradation, because the recombinant strain without the *mlrD* gene can also degrade MCLR and excrete the intermediate product (linear MCLR) out of cells. This phenomenon has also been found in other studies [13,30]. Hence, the function of *mlrD* in MCs degradation needs further research.

The *mlr* cluster in strain X20 had a total length of 5575 bp and a G + C content of 59.05%, in which *mlrA*, *mlrB*, *mlrC*, and *mlrD* were contained with the full length of 1011 bp, 1626 bp, 1587 bp, and 1272 bp, respectively. The four *mlr* genes in strain X20 have the same order and translation orientation as that in strain ACM-3962 [7]. Up to date, three full sequences of the *mlr* gene cluster and one partial sequence have been reported, which are from *Sphingopyxis* sp. C-1, *Sphingosinicella* sp. B-9, *Novosphingobium* sp. THN1, and *Sphingomonas* sp. ACM-3962, respectively. The four *mlr* cluster sequences have high similarity to that in strain X20 (86.8–98.1%), suggesting that they may come from a similar ancestor gene. It is noteworthy that most of the *mlr* sequences reported so far are from α-proteobacteria, especially from sphingomonads. Except for sphingomonads, no MC-degrading ability or *mlr* genes have been identified in other Sphingomonadaceae to date, although *mlr* genes have been found in a species of Rhizobiales [13] and two species of β-proteobacteria [17] and γ-proteobacteria [31]. The reason for the concentrated distribution of MC-degrading ability and *mlr* genes in sphingomonads is still unclear. The information on the evolutionary origin of *mlr* genes may facilitate the clarification of this phenomenon.

### 2.3. Evolutionary Origin of mlr Gene Cluster

**Figure 3:**
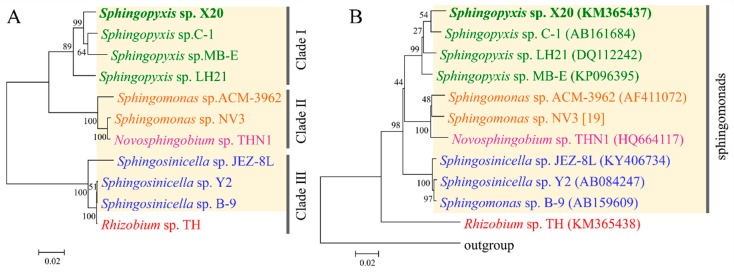
Phylogenetic tree inferred from spliced *mlr* gene sequences (**A**) and 16S rDNA (**B**) from the same set of microcystin (MC)-degrading bacteria. Evolutionary analyses were conducted by the neighbor-joining method in MEGA7. The numbers at each node were the bootstrap values for the percentages of 1000 replicate trees.

Although the evolutionary origin of *mlrA* has been elucidated in some detail, the origin of three other *mlr* genes, especially *mlrB* and *mlrC*, remains unknown [13,14]. Since the enzymes encoded by the two genes are also critical for MCs decomposition, it is necessary to understand their evolutionary origin [7]. To verify whether these genes co-evolved with *mlrA*, phylogenetic trees were constructed using the spliced *mlr* sequences by ML, NJ, and ME methods, respectively. The same topology was found in the trees constructed by the NJ and ME methods (Figure 3A). As shown in Figure 3A, all the species were divided into three clades. The *Sphingopyxis* species formed a clade (clade I), while the *Sphingomonas* species formed another tight clade (clade II) with a *Novosphingobium* species. The two subclades composed a major clade, and another main clade consisted of the *Sphingosinicella* and *Rhizobium* species (Figure 3A). In a phylogenetic tree based on protein sequences translated from the nucleotide sequences of the spliced *mlr*, all the species occupied the same phylogenetic positions as those in the *mlr* tree except for *Sphingopyxis* sp. LH21, which did not cluster with the other *Sphingopyxis* species (Figure S1). Moreover, a similar topology was also observed for analyses performed separately with the four *mlr* genes (data not shown). The topological congruence indicated that the four *mlr* genes might have the same origin and evolutionary history.

**Figure 4:**
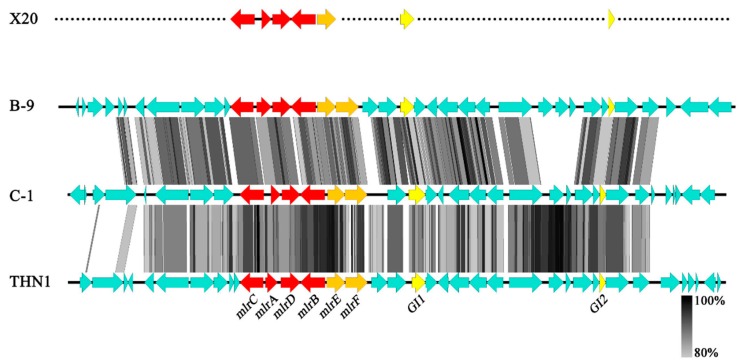
Comparison of the conserved region on the genomic islands (GIs) of *Sphingosinicella* sp. B-9 (AP018711), *Sphingopyxis* sp. C-1 (NZ_BBRO00000000), and *Novosphingobium* sp. THN1 (CP028347). Genomic comparisons were performed using BLASTn, with a maximum e-value of 0.001 and a minimum hit length of 20 bp. The figure was produced using Easyfig v2.2.3. Predicted genes and the direction of transcription were notated by block arrows. The grey-black region indicates the sequence similarity (from 80% to 100%). The corresponding genes in strain X20 were also noted.

GI analyses provided further evidences for the co-evolution of the four *mlr* genes. Only three genomes of MC-degrading bacteria (*Sphingopyxis* sp. C-1, NZ_BBRO00000000; *Sphingosinicella* sp. B-9, AP018711; and *Novosphingobium* sp. THN1, CP028347) have been sequenced so far, and they are all from sphingomonads. GI analyses showed that there was a GI of 60.3 kb within the strain C-1 genome, on which the *mlr* gene cluster anchored. A similar GI of 130.0 kb was also found on the strain B-9 genome, inside which a shorter GI (18.2 kb) with the *mlr* gene cluster was nested. Although the similar GI was not found in strain THN1, a highly similar DNA region to the GI (about 33 kb, with similarities of 88.7% and 86.4% to strain C-1 and B-9) was found on the genome, with *mlr* genes on it. Therefore, it is likely that strain THN1 also possessed a similar GI, and the failure of detection might be due to the loss or rearrangement of some sequence regions during its evolution. Further BLAST analyses found no similar GI or sequence on the genomes of other sphingomonads without MC-degrading ability. Moreover, many genes on the GI were specific to the three strains, whereas the genes adjacent to the GI were also found on the genomes of other sphingomonads currently available, independent of their MC-degradation ability. These results implied that the GI is likely unique to the MC-degrading bacteria with the *mlr*-dependent pathway. To test the hypothesis, four genes (*mlrE*, *mlrF*, *GI1,* and *GI2*) on the GI, which were unique to the three MC-degrading bacteria, were selected as the marker genes to determine whether strain X20 possessed the same GI (Figure 4). Three of the four genes—*mlrE*, *GI1*, and *GI2*—were successfully amplified and sequenced. The three genes in strain X20 share very high similarity (86.0–98.6%) with those from strains C-1, B-9, and THN1, suggesting that similar GI may also be present in strain X20. The reason for failure in *mlrF* amplification is still unclear. The mismatch between primers and template might be one of the possible reasons, since only three *mlrF* sequences were reported to date, which exacerbated the difficulty of designing appropriate primers.

Since the genomes have not been reported for most MC-degrading bacteria, it is still unknown whether the *mlr* genes anchor on the similar GIs or conserved regions in these species. However, our results suggested that *mlr* gene clusters have likely entered into the genome of sphingomonads by horizontal gene transfer of the GI, and then evolved together with it, since the G + C contents (60.8% and 60.1%) of the GIs were significantly lower than that (63.7% and 63.9%) of associated genomes, but near to that (59.0% and 59.1%) of the *mlr* gene clusters in *Sphingopyxis* sp. C-1 and *Sphingosinicella* sp. B-9. That the *mlrE* and GI2 trees have identical topological properties with the *mlr* tree for strains C-1, B-9, THN1, and X20 (data not shown) provided further evidence for this hypothesis. In addition, despite the relatively concentrated distribution of the *mlr*-dependent pathway in sphingomonads, not all species of sphingomonads possess these genes. This phenomenon also agrees well with the above deduction.

**Figure 5:**
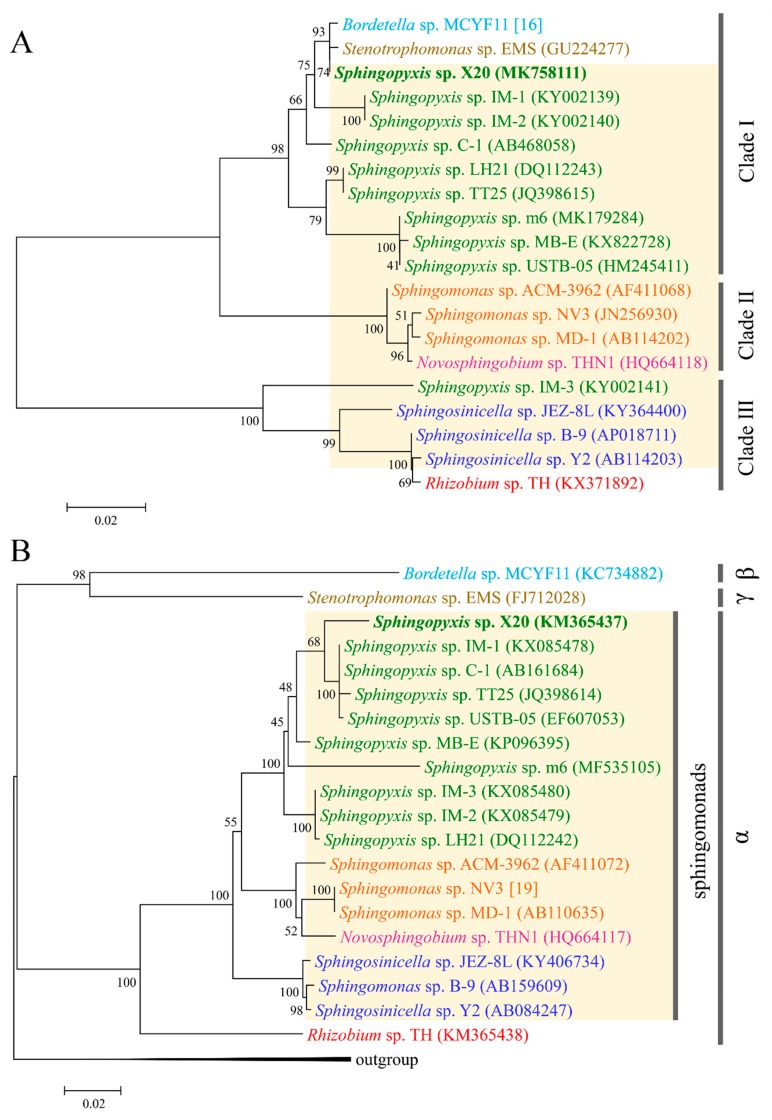
Phylogenetic tree of the *mlrA* gene (**A**) and 16S rDNA (**B**) from the same set of MC-degrading bacteria. Evolutionary analyses were conducted by the neighbor-joining method in MEGA7. The numbers at each node were the bootstrap values for the percentages of 1000 replicate trees. The Greek letters denoted α-proteobacteria, β-proteobacteria, and γ-proteobacteria, respectively.

To further clarify the evolutionary origin of the *mlr* gene cluster, 16S rDNA trees were constructed using the dataset from the same strains. Our results showed that the trees constructed by the NJ, ML, and ME methods shared the same topology. Furthermore, the 16S rDNA tree had a similar pattern with the *mlr* tree for sphingomonads (Figure 3A,B). In the two trees, all the sphingomonads formed three clades with taxonomically closer species clustering together. The congruent topology indicated that the *mlr* gene clusters in various genera of sphingomonads may originate from a single ancestor gene, rather than from recent horizontal gene transfer. Considering that limited *mlr* clusters data may play a role in the congruence, a comparison between the *mlrA* tree and 16S rDNA tree was also conducted by using currently available *mlrA* genes and the associated 16S rRNA genes. The same topology was obtained for sphingomonads species in both *mlrA* trees and 16S rDNA trees constructed by the NJ, ML, and ME methods (Figure 5A,B). The same topology was also observed in the phylogenetic tree inferred from MlrA protein sequences (Figure S2). The topological congruence provided further support for the above proposition. Currently, most α-proteobacteria containing *mlr* genes were from sphingomonads, which is composed of closely related genera, and only a *Rhizobium* sp. strain TH was from the order Rhizobiales. The broad distribution of *mlr* gene cluster in sphingomonads degraders suggested that these genes have been present in the sphingomonads gene pool for a considerable time. These findings implied that the *mlr* gene cluster together with a GI probably has been acquired very early in the evolution of sphingomonads by a horizontal gene transfer event, and then some species of sphingomonads gained it through vertical inheritance. Therefore, the acquisition of GI with the *mlr* gene cluster is likely a key step in the evolution of the *mlr*-dependent pathway.

As for *Rhizobium* sp. strain TH, the origin of *mlr* is likely different from that of sphingomonads. Strain TH tightly clustered with *Sphingosinicella* species in both *mlrA* and *mlr* trees (Figure 5A and Figure 3A), rather than formed a distinct clade, although it belongs to the order Rhizobiales, which is taxonomically distinct from sphingomonads (Figure 5B and Figure 3B). This result disagreed with that reported previously [13]. The divergence might be due to the limited dataset in the reference [13]. Considering the concentrated distribution of *mlr* gene clusters among sphingomonads, the high homology of *mlr* gene clusters between strain TH and *Sphingosinicella* species suggested that strain TH possibly acquired the *mlr* gene cluster from *Sphingosinicella* by recent lateral gene transfer. Although the *mlrA* gene has been detected and sequenced in a β-proteobacterial isolate (*Bordetella* sp. MCYF11) and a γ-proteobacterial isolate (*Stenotrophomonas* sp. EMS) [17,31], the *mlr* gene clusters have not been sequenced to date. Hence, the evolutionary origin of the *mlr* cluster is still unknown for Proteobacteria other than α-proteobacteria. Nevertheless, the high homology of the *mlrA* gene with *Sphingopyxis* species (Figure 5A) and the sporadic distribution of *mlr* genes among these species indicated that these MC-degrading bacteria might obtain the *mlr* gene cluster from *Sphingopyxis* by recent lateral gene transfer.

Although GIs are widespread in bacterial genomes, it is the first time, to our knowledge, that a GI containing an *mlr* gene cluster was reported. Acquisition of the GI enabled sphingomonads to expand its genome to exploit new environmental niches and may provide them with a competitive advantage over other species during water blooms. This may be one of the reasons that most of the MC-degrading bacteria with an *mlr*-dependent pathway are from sphingomonads. Up to now, only part of the *mlr* pathway for MC degradation has been clarified; other major genes, especially those involved in Adda degradation, have not been elucidated. Since the *mlr* gene cluster may have co-evolved with the GI, it is likely that other main genes involved in MC degradation are present on it [32,33]. The analyses of the GI may help discover new genes within this pathway and contribute to the clarification of the whole MC-degrading process.

## 3. Conclusions

An MC-degrading bacterium strain X20 was isolated from Dianchi Lake and identified as *Sphingopyxis* sp. The complete *mlr* gene cluster sequence of strain X20 was obtained and the activity of *mlrA* gene was verified by heterologous expression. Phylogenetic analysis and genomic island analyses suggested that the four *mlr* genes had the same origin and evolutionary history. The *mlr* gene cluster may has initially entered into the genome of sphingomonads by the horizontal gene transfer of a genomic island and then was inherited by some sphingomonads. Thereafter, the species other than sphingomonads obtained it by recent horizontal gene transfer.

## 4. Materials and Methods

### 4.1. Materials and Reagents

The MCLR standard was purchased from Sigma-Aldrich (St. Louis, MO, USA). The MCLR for biodegradation experiments was extracted and purified from laboratory-cultured *Microcystis aeruginosa* PCC 7806, as described previously [34]. Methanol and trifluoroacetic acid (TFA) (Tedia Company, Inc., Fairfield, OH, USA) used as the mobile phase of high-performance liquid chromatography (HPLC) were of HPLC grade. Other chemicals were of analytical grade.

### 4.2. Isolation and Identification of MC-Degrading Bacterium

Surface sediment was sampled from Dianchi Lake in China. The isolation and identification of MC-degrading bacterium were performed as reported previously [13]. Briefly, after the enrichment with MCLR-containing mineral salt medium (MSM), individual colonies were isolated from sediment by serial dilution in MSM and subsequent isolation on solid media. The MC-degrading ability was detected in MCLR-containing MSM by high-performance liquid chromatography (HPLC). One isolate with high MC-degrading ability was isolated and named as X20. The 16S rDNA of strain X20 was amplified and sequenced, and the sequencing data have been deposited in GenBank under accession number KM365437. Based on the sequences of strain X20 and related type strains in GenBank, a phylogenetic tree was constructed using the neighbor-joining method in MEGA7.0.

### 4.3. MCLR Degradation Experiments

Strain X20 was incubated overnight in yeast extract-peptone medium (YPM) (containing yeast extract 3 g, peptone 3 g, MgSO_4_·7H_2_O 0.5 g, and CaCl_2_ 0.3 g per liter) on a shaker (120 rpm) at 30 °C for enrichment. The enriched cells were centrifuged at 8000 rpm for 5 min and washed with MSM twice. The cells were resuspended in MSM containing MCLR (approximately 5 mg·L^−1^) and cultivated at 30 °C. The same culture medium without bacterial inoculum was used as a control. Samples were collected from the cultures at regular intervals. The concentration of MCLR was monitored by HPLC, and the bacterial growth was measured via detection of the absorbance at 600 nm (OD600). All the experiments were conducted in triplicate.

### 4.4. Sequencing of the mlr Gene Cluster

The partial sequence of the *mlr* gene cluster in strain X20 was obtained by amplification of regions spanning *mlrC*-*mlrA*, *mlrA*-*mlrD*, and *mlrD*-*mlrB*, as described previously [13]. The flanking regions of the *mlr* gene cluster were obtained by thermal asymmetric interlaced (TAIL)-PCR. Two groups of nested insertion-specific primers for TAIL-PCR (Table 1) were designed based on the *mlrC* and *mlrB* partial sequences of strain X20. TAIL-PCR was performed with the Genome Walking kit (Takara) according to the manufacturer’s instructions. All the PCR products above were sequenced and assembled to obtain a full-length *mlr* gene cluster, which has been deposited in the GenBank database (accession no. MK758111). Comparison with other *mlr* gene clusters were conducted by BLAST.

### 4.5. Heterogeneous Expression of the mlrA Gene

To verify the activity of the *mlr* gene cluster, primers were designed based on the *mlrA* sequence of strain X20, with *BamHI* and *Hind*III sites added to the forward and reverse primers (Table 1). The heterogeneous expression of *mlrA* gene was performed in *E. coli* BL21 (DE3) and the activity of recombinant strains and recombinant enzyme were detected as reported previously [13]. The concentrations of MCLR and its intermediate products were determined by comparing retention times under the same HPLC conditions with that of standard MCLR or intermediate products previously reported [13].

### 4.6. Phylogenetic Analyses

Due to the lack of a whole *mlr* gene cluster, a spliced sequences dataset was assembled by the sequences of four *mlr* genes from each isolate for the construction of a phylogenetic tree. Each of the four *mlr* genes was retrieved from GenBank (Table S1) and aligned and trimmed by clustalX 2.1 to the longest fragment available, respectively. The segments of *mlrA*, *mlrB*, *mlrC,* and *mlrD* with 700 bp, 335bp, 546 bp, and 539 bp, respectively, were obtained and assembled to form a set of 2120 bp-spliced sequences. Phylogenetic trees were inferred using both the segments and the spliced sequences datasets by the maximum-likelihood (ML), minimum-evolution (ME), and neighbor-joining (NJ) methods in MEGA7. A phylogenetic tree was also constructed based on 16S rDNA sequences from the same sets of taxa by using *Desulfobacter halotolerans* DSM 11383^T^ (NR_026439) as an outgroup. In contrast to the limited number of sequences for three other genes, more *mlrA* sequences have been reported. Therefore, a phylogenetic tree was inferred using the *mlrA* sequences currently available to further clarify the evolutionary origin of *mlr* genes. A 16S rDNA tree was also constructed by using *Bacillus cereus* ATCC 14579^T^ (MH281748) and *Arthrobacter globiformis* DSM 20124^T^ (NR_026187) as outgroups. Phylogenetic trees were also constructed based on protein sequences translated from the nucleotide sequences of the spliced *mlr* or *mlrA* genes, respectively.

### 4.7. Genomic Island Analyses

The genome sequences of three MC-degrading bacteria—*Sphingopyxis* sp. C-1 (NZ_BBRO00000000), *Sphingosinicella* sp. B-9 (AP018711), and *Novosphingobium* sp. THN1 (CP028347)—were retrieved from GenBank. The genomic islands (GIs) on the genomes of these strains were screened by the IslandViewer 4 web server with default settings [35]. The islands detected were compared with each other by BLAST to identify the conserved region, which was then used to search the genomes of sphingomonads by BLAST for similar sequences. The genes on the GIs and adjacent to the GIs were also used to search the GenBank database by BLAST.

To verify the existence of a similar GI in strain X20, two genes (*mlrE* and *mlrF*) previously reported [33] and two conserved genes (named as *GI1* and *GI2*) on the GIs were chosen as markers. The primers were designed according to the conserved regions in the three strains (strain C-1, B-9, and THN1) (Table 1). The PCR reaction was performed with the genomic DNA of strain X20 as a template, and the PCR conditions were as follows: 94 °C for 5 min followed by 30 cycles of 94 °C for 20 s, 55 °C for 15 s and 72 °C for 40 s; then, 72 °C for 10 min. The amplified products of three genes (*mlrE*, *GI1*, and *GI2*) were sequenced after purification, and the comparisons of them among various strains were performed by BLAST. The phylogenetic trees were also inferred by the NJ method using the three genes, respectively.

## Figures and Tables

**Table 1:** Primer sequences used in this study.

Gene	Primer	Sequence (5′–3′)	Purpose	References
*mlrC*-*mlrA*	mlrCf1	TCCCCGAAACCGATTCTCCA	Partial *mlr*	[21]
	MR	CTCCTCCCACAAATCAGGAC		[23]
*mlrA*-*mlrD*	MF	GACCCGATGTTCAAGATACT	Partial *mlr*	[23]
	mlrDr1	ACAGTGTTGCCGAGCTGCTCA		[21]
*mlrD*-*mlrB*	mlrDf1	GCTGGCTGCGACGGAAATG	Partial *mlr*	[21]
	mlrBr1	CGTGCGGACTACTGTTGG		
*mlrB*	mlrBf2	ATGACTGCAACAAAGCTTTT	Partial *mlr*	This study
	mlrBr2	TTATCCACGAACAACCCACC		
*mlrC*	CR1	CCCTGGCAGTACAATTGGGCTTTGA	Flanking region	This study
	CR2	CACAGGGCTTGCCGAGAATGTCA		
	CR3	CGTCAGCGAAATTCGCGACCAGT		
*mlrB*	BF1	AGGTAGGTCAGGCAGATAGGTG	Flanking region	This study
	BF2	AAGATCAGGATGAGAACGGCCG		
	BF3	AGATCAGCAAGTCCAAAGCCGC		
*mlrA*	MlrAxf	GACGGATCCATGCGGGAGTTTGTCAAAC	Expression	This study
	MlrAxr	TATAAGCTTCGCGTTCGCGCCGGACTTG		
*mlrE*	mlrEf	TTCGGTAGACGGAACACA	GI verification	This study
	mlrEr	ACACGGCATTGATCTGAAT		
*mlrF*	mlrFf	GATGGAAGAGGTGATGGCAATT	GI verification	This study
	mlrFr	AGGACGAATACTGGTGGTAGTC		
*GI1*	G1f	ACTCTGGACCAGCGGCTAA	GI verification	This study
	G1r	CAAGCGGACTGACAAGTTCTG		
*GI2*	G2f	GCAACCGTCATCAGTGGATC	GI verification	This study
	G2r	CCGCCGTAGTATTCGTGAATG		

## Supplementary Materials

The following are available online at https://www.mdpi.com/2072-6651/11/5/269/s1, Figure S1: Phylogenetic tree inferred from protein sequences of spliced mlr sequences. Evolutionary analysis was conducted by Neighbor-Joining method in MEGA7. The numbers at each node were the bootstrap values for the percentages of 1000 replicate trees, Figure S2: Phylogenetic tree based on MlrA protein sequences. Evolutionary analyses were conducted by the Neighbor-Joining method in MEGA7. The numbers at each node were the bootstrap values for the percentages of 1000 replicate trees. * represent the protein sequence translated from the nucleotide sequence of the *mlrA* gene, Table S1: Sequences used to construct assembled mlr gene cluster.
