# Supplementary material for: Isolation of a Novel Microcystin-Degrading Bacterium and the Evolutionary Origin of mlr Gene Cluster
Source: Toxins (Basel). 2019 May 13;11(5):269. doi: 10.3390/toxins11050269 (PMC6563193; doi:10.3390/toxins11050269)
Supplement: Supplementary file 1 [file toxins-11-00269-s001.pdf]

Supplementary Materials: Isolation of a novel microcystin-degrading bacterium and the evolutionary origin of *mlr* gene cluster

Lian Qin, Xiaoxing Zhang, Xiaoguo Chen, Ke Wang, Yitian Shen and Dan Li

Table S1. Sequences used to construct assembled *mlr* gene cluster.

Bacteria	GenBank accession no.			
	mlrA	*mlrB*	*mlrC*	*mlrD*
Sphingopyxis sp. C-1	AB468058	AB468059	AB468060	AB591070
Sphingopyxis sp. LH21	DQ112243	DQ423530	DQ423531	DQ423532
Sphingopyxis sp. MB-E	KX822728	KX822729	KX822730	KX822731
Sphingomonas sp. ACM-3962	AF411068	KR150744	AF411070	AF411071
Sphingomonas sp. NV3	JN256930	JN256929	JN256928	JN256927
Sphingosinicella sp. Y2	AB114203	KY002145	KY002149	KY002153
Sphingosinicella sp. JEZ-8L	KY364400	KY364403	KY364402	KY364401
Sphingopyxis sp. X20	MK758111			
Novosphingobium sp. THNI	HQ664118			
Rhizobium sp. TH	KX371892			
Sphingosinicella sp. B-9	AP018711*			

* Genome sequence.

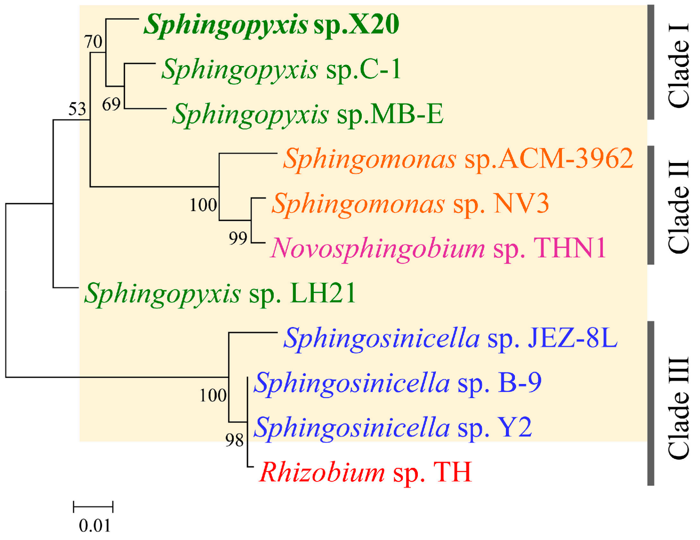

Figure S1. Phylogenetic tree inferred from protein sequences of spliced *mlr* sequences. Evolutionary analysis was conducted by Neighbor-Joining method in MEGA7. The numbers at each node were the bootstrap values for the percentages of 1000 replicate trees.

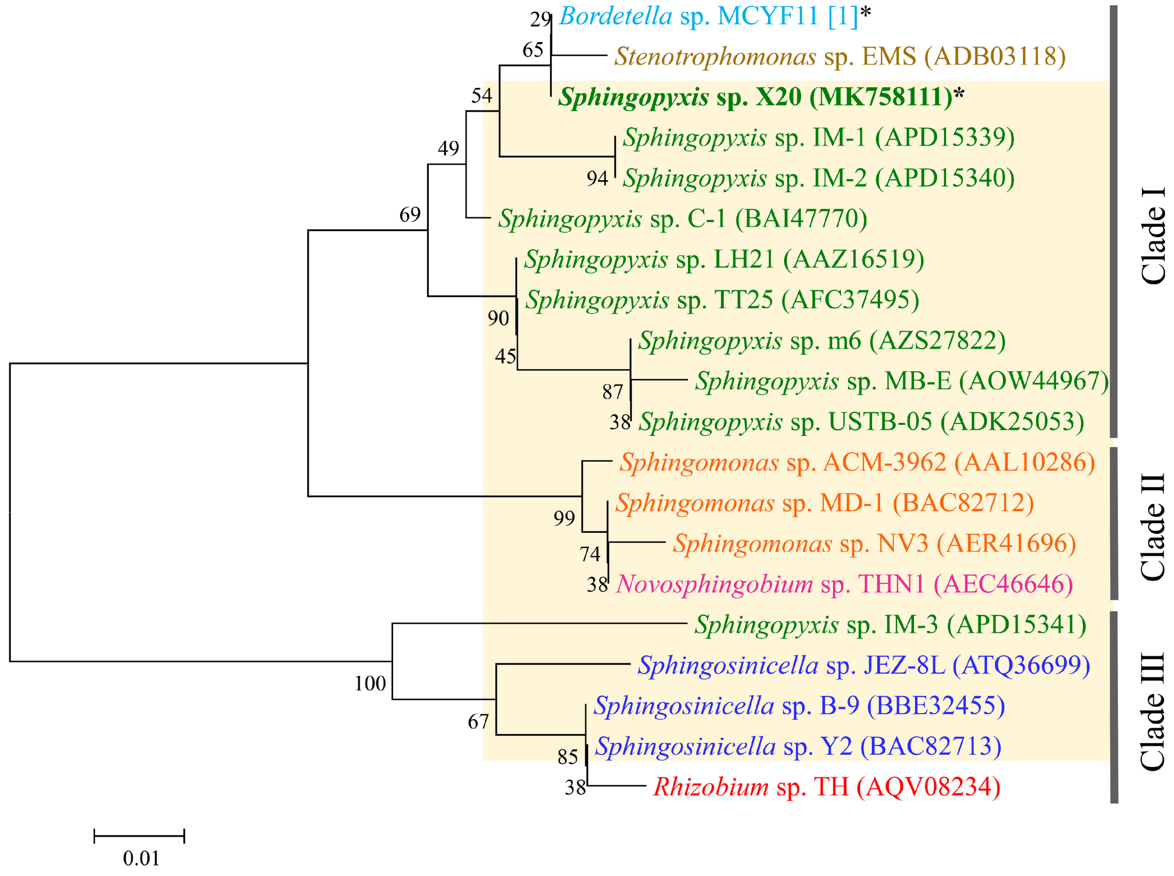

Figure S2. Phylogenetic tree based on MlrA protein sequences. Evolutionary analyses were conducted by the Neighbor-Joining method in MEGA7. The numbers at each node were the bootstrap values for the percentages of 1000 replicate trees. * represent the protein sequence translated from the nucleotide sequence of the *mlrA* gene.

References

1. Yang, F.; Zhou, Y.; Sun, R.; Wei, H.; Li, Y.; Yin, L.; Pu, Y. Biodegradation of microcystin-LR and-RR by a novel microcystin-degrading bacterium isolated from Lake Taihu. *Biodegradation* **2014**, *25*, 447–457, doi:10.1007/s10532-013-9673-y
